# Effect of Antioxidant Supplementation on NET Formation Induced by LPS In Vitro; the Roles of Vitamins E and C, Glutathione, and N-acetyl Cysteine

**DOI:** 10.3390/ijms241713162

**Published:** 2023-08-24

**Authors:** Germán Muñoz-Sánchez, Lucila A. Godínez-Méndez, Mary Fafutis-Morris, Vidal Delgado-Rizo

**Affiliations:** 1 Departamento de Fisiología, Centro Universitario de Ciencias de la Salud, Universidad de Guadalajara, Guadalajara 44340, Mexico; german.munoz2599@alumnos.udg.mx (G.M.-S.); ariadnegodinez@gmail.com (L.A.G.-M.); 2 Centro de Investigación en Inmunología y Dermatología (CIINDE), Calzada Federalismo Norte #3102, Zapopan 45190, Mexico

**Keywords:** antioxidants, neutrophil extracellular traps, reactive oxygen species, reactive nitrogen species

## Abstract

Neutrophil extracellular traps (NETs) require reactive oxygen species (ROS) to eliminate pathogens by inducing oxidative stress. However, this process can also cause tissue damage to the host. Neutrophils contain high concentrations of vitamin C (1.5 mM) compared to the bloodstream (0.1 mM), and this antioxidant can interact with vitamin E and glutathione (GSH) inside the cell to maintain the redox balance. Previous studies have investigated the effect of vitamins E or C and N-acetyl cysteine (NAC) on NET formation, but the interactions of these molecules in neutrophils remain unknown. In this study, we investigated the effect of antioxidants alone and two combinations on NET formation and oxidative stress. Neutrophils were pre-loaded with GSH + NAC or vitamin E + vitamin C + GSH + NAC (termed ALL), and LPS-induced NET formation was assessed using fluorometry and immunofluorescence. Antioxidant effects were evaluated by measuring the total antioxidant capacity (TAC), GSH/GSSG ratio, ROS production, nitrite + nitrate levels, and lipid peroxidation. Our results showed that even low doses of antioxidants are capable of decreasing NETs. Furthermore, the combinations augmented TAC and GSH/GSSG ratio and decreased ROS, nitrites + nitrates, and malondialdehyde (MDA) levels in supplemented neutrophils in vitro.

## 1. Introduction

Neutrophils are cells of the innate immune system that make up over 60% of the total white blood cells in the bloodstream. They are the first line of defense against invading pathogens such as bacteria, fungi, and viruses [1,2,3]. NETs are defense mechanisms of neutrophils that form web-like structures composed of chromatin and antimicrobial molecules that are released to trap and kill invading pathogens [4]. Excessive NET formation has been linked to harmful effects in the host [5,6,7]. As a result, various investigations have focused on uncovering the regulatory mechanisms of NETs.

During NET formation, reactive oxygen species (ROS) production is crucial for pathogen elimination [8], but it can also cause oxidative stress and host damage by reacting with intracellular and extracellular molecules such as phospholipids, proteins, and DNA [9,10].

Neutrophil activation leads to the assembly of the NADPH complex, which serves as the primary source of ROS. Upon assembly, the NADPH complex generates superoxide anion (O_2_^●−^), which is subsequently converted to hydrogen peroxide (H_2_O_2_) by the action of the superoxide dismutase enzyme (SOD). H_2_O_2_ then interacts with intracellular Fe^+2^ or Cu^+2^, resulting in the formation of the hydroxyl radical (OH^−^) [11,12,13]. Once neutrophils undergo NETosis, their ability to generate ROS is null. Nitric oxide (NO) is the main reactive nitrogen species (RNS) involved in NET formation, generating peroxynitrite (ONOO^−^) and inducing protein nitration [14,15].

The role of ROS in NET formation has been extensively studied, and it has been demonstrated that ROS is necessary for effective NET release. In diseases with alterations in ROS production in neutrophils, such as chronic granulomatous disease (CGD), the process of NET formation is impaired, rendering the individuals more susceptible to recurrent infections [16]. In patients with myeloperoxidase (MPO) deficiency, the interaction between H_2_O_2_ and MPO is disrupted, and the antimicrobial activity of neutrophils is defective [17,18,19,20]. These findings highlight the crucial involvement of ROS and suggest the existence of a mechanism that regulates redox homeostasis in healthy individuals and governs NET formation.

Organisms have developed exogenous and endogenous antioxidants to counteract the harmful effects of free radicals [11,21]. Vitamins E and C are exogenous antioxidants obtained through the diet that can interact with each other, scavenge ROS, and protect against oxidative stress [22,23,24,25], whereas glutathione (GSH) is an endogenous antioxidant synthesized inside cells that can reduce oxidized vitamin C [26,27]. Exogenous and endogenous antioxidants can interact with each other, improving the antioxidant capacity of cells. These interactions depend on the redox potential, chemical nature, and localization of each molecule (Table 1).

Human concentrations of these vitamins have been described. For vitamin E, serum levels range from 22 to 28 µM [29]. Vitamin C levels have been characterized in many conditions in humans: deficient levels (0.003 mM), hypovitaminosis (0.03 mM), normal serum levels (0.1 mM), intracellular levels in neutrophils, and pharmacological levels (1–3 mM) [30,31,32]. It has been reported that plasma levels of GSH in healthy subjects are 0.8 mM, while most cells present higher concentrations ranging from 2 to 10 mM depending on their metabolic activity [27]. Studies have revealed that GSH levels can increase through the supplementation of NAC either orally or intravenously [35,36]. Moreover, commercial formulations, such as the University of Wisconsin Solution (UWS) for organ preservation, contain quantities similar to those reported inside the cell, including 2–3 mM [37].

Previous studies have shown that antioxidants can suppress NET formation induced by phorbol myristate acetate (PMA). For instance, vitamin C and NAC can reduce both NET formation and ROS, while α-tocopherol appears to have no effect on NET formation but can reduce ROS [31,32]. Another study performed by Sae-Khow et al. with neutrophils from septic patients observed that low concentrations of ascorbate (1 mM) decreased spontaneous NETosis to that of normal healthy volunteers, while high concentrations of ascorbate (≥10 mM) promoted NET formation [38]. There is no information on the effect of these vitamins in human neutrophils stimulated with a natural stimulus such as lipopolysaccharide (LPS), a stimulus that is commonly present in the membrane of Gram-negative bacteria [39]. In addition, the synergistic effects of the vitamins and other antioxidants, such as GSH and NAC, have not been tested in NET formation assays.

Our investigation aims to clarify the effect of antioxidants alone: vitamins E and C, GSH, and NAC at reported concentrations in humans on NET formation induced by LPS in vitro. We also aim to demonstrate the effects of antioxidant combinations, including GSH + NAC and ALL antioxidants, on LPS-stimulated neutrophils. This includes TAC, redox state, ROS and RNS production, and lipid peroxidation levels. Our results demonstrate that supplementing human neutrophils alone or in combination reduces LPS-induced NET formation. Furthermore, the GSH + NAC and ALL antioxidant supplementation of neutrophils augment the TAC of neutrophils, improve the redox state, decrease ROS and RNS, and prevent lipid peroxidation.

## 2. Results

### 2.1. Different Doses of Vitamins C and E, GSH, and NAC Reduce NETs in a Concentration-Dependent Manner

Neutrophils were incubated with or without antioxidants for 60 min, followed by stimulation with LPS (10 µg/mL). Our results showed that all of the antioxidants induced a concentration-dependent suppressive effect on NET formation.

**Figure 1:**
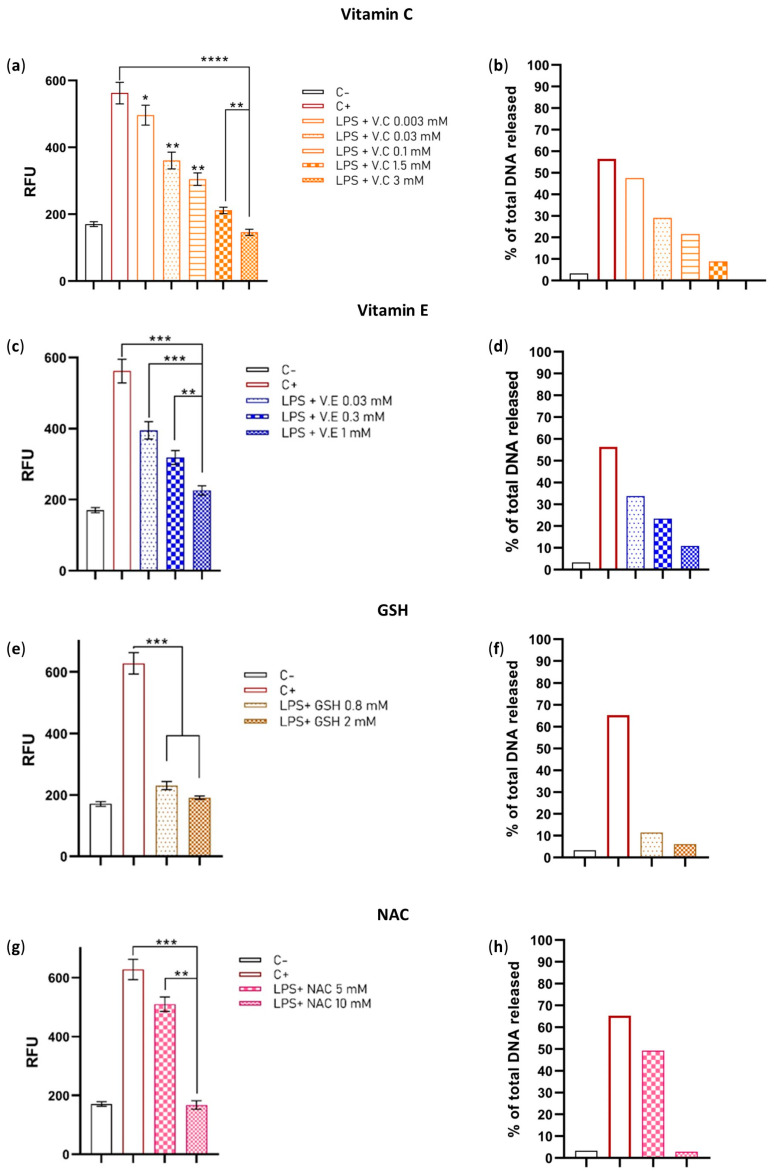

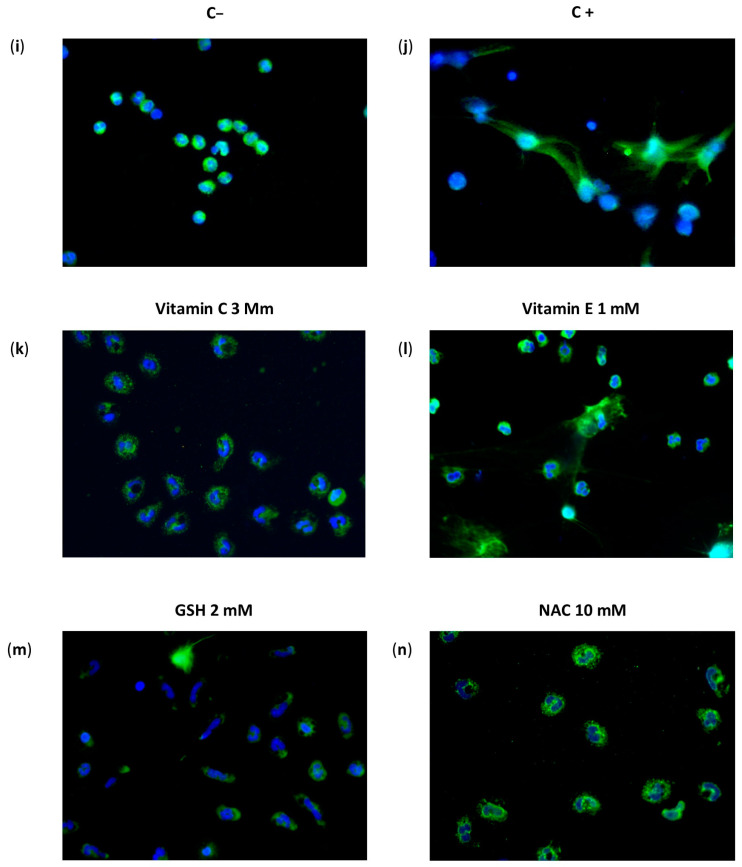
Effect of the antioxidants, vitamin C, vitamin E, GSH, and NAC, on NET formation induced by LPS. (**a**) NET inhibition with the concentrations of vitamin C used: insufficient (0.003 mM), deficient (0.03 mM), normal (0.1 mM), inside the neutrophil (1.5 mM), and pharmacological (3 mM). (**b**) Percentage of DNA released by vitamin C-supplemented neutrophils at different concentrations. (**c**) NET inhibition with the concentrations of vitamin E used: (0.003 mM) serum level, (0.03 mM) adipocyte level, and (1 mM) experimental on NETs. (**d**) Percentage of DNA released by vitamin E-supplemented neutrophils at different concentrations. (**e**) NET inhibition with the concentrations of GSH used: (0.8 mM) serum levels and (2 mM) intracellular levels. (**f**) Percentage of DNA released by GSH-supplemented neutrophils at two different concentrations. (**g**) NET inhibition with the concentrations of NAC used: 5 mM and 10 mM. (**h**) Percentage of DNA released by NAC-supplemented neutrophils at two different concentrations. (**i**) Fluorescence microscopy of the negative control (C−). (**j**) Fluorescence microscopy of the positive control (C+). (**k**) Fluorescence microscopy of the neutrophils treated with 3 mM of vitamin C. (**l**) Fluorescence microscopy of the neutrophils treated with 1 mM of vitamin E. (**m**) Fluorescence microscopy of the neutrophils treated with 2 mM of GSH. (**n**) Fluorescence microscopy of the neutrophils treated with 10 mM of NAC. The sample size was 5 subjects for each triplicate. Data were normalized by inverse function and one-way ANOVA, and T3-Dunnet post hoc test was performed. Results are presented as relative fluorescent units (RFU) mean ± standard error (S.E.) (**a**,**c**,**e**,**g**) and percentage of NETs (**b**,**d**,**f**,**h**). Significant *p*-values: <0.03 (*), <0.002 (**), <0.001 (***), <0.0001 (****). Immunofluorescence was performed using DAPI for DNA staining and anti-NE for neutrophil elastase granules at 40× magnification (**i**–**n**); each image is representative of four independent experiments.

At a deficient concentration of 0.003 mM, vitamin C had a significant suppressive effect when compared to the positive control. Furthermore, we observed that its effect improved with increasing concentrations of 0.03 mM (hypovitaminosis), 0.1 mM (serum level), 1.5 mM (neutrophil intracellular), and 3 mM (pharmacological) (Figure 1a,b). The highest concentration of vitamin C exerted the strongest effect and was significantly better than the other concentrations. Fluorescent microscopy revealed the absence of NETs was observed in pre-loaded neutrophils with 3 mM of vitamin C (Figure 1k). These findings demonstrate that vitamin C can decrease NET formation even at deficient concentrations in human blood. Moreover, at pharmacological concentrations, it can completely prevent NET formation. This is the first report to demonstrate that deficient concentrations of vitamin C can decrease NET formation.

Vitamin E showed similar results, with concentrations of 0.03 mM (serum level), 0.3 mM (adipocyte membrane concentration), and 1 mM (experimental concentration) significantly decreasing NETs in a concentration-dependent manner (Figure 1c,d). Additionally, the highest concentration (1 mM) showed an effect consistent with the quantitative result in fluorescent microscopy (Figure 1l). Our findings demonstrate that the serum concentration of vitamin E can control NET formation, and at higher concentrations (10 times; 0.3 mM or 33 times; 1 mM of the serum level), we can decrease NETs to near basal levels.

In turn, GSH at a concentration of 0.8 mM (serum level) significantly reduced NET formation, and the effect was slightly improved when a concentration of 2 mM was used (Figure 1e,f). Fluorescent microscopy showed consistent results, with NETs reduced to near basal levels (Figure 1m). These findings demonstrate that serum and intracellular levels of GSH have the same effect on decreasing NET formation. Unlike the last two antioxidants, this is the first time that GSH has been used to study its effect on NET formation.

Finally, we used two different concentrations of NAC that were previously used by Kirchner et al. in 2013. Our results showed that a concentration of 5 mM was capable of significantly decreasing NET formation. Furthermore, when the concentration was increased to 10 mM, the effect was significantly improved (Figure 1g,h), and no NETs were detected when observed with fluorescent microscopy (Figure 1n).

This experiment demonstrates that vitamins C and E, GSH, and NAC can control NET formation in vitro at different levels depending on the concentration used. Furthermore, we found that high concentrations of these antioxidants can prevent NET formation to near basal levels, suggesting that redox balance is important in the context of NETs. However, it is important to note that antioxidants may be able to interact with each other and improve their effect.

### 2.2. The Combination of GSH + NAC and ALL Antioxidants Completely Abolishes NET Formation

**Figure 2:**
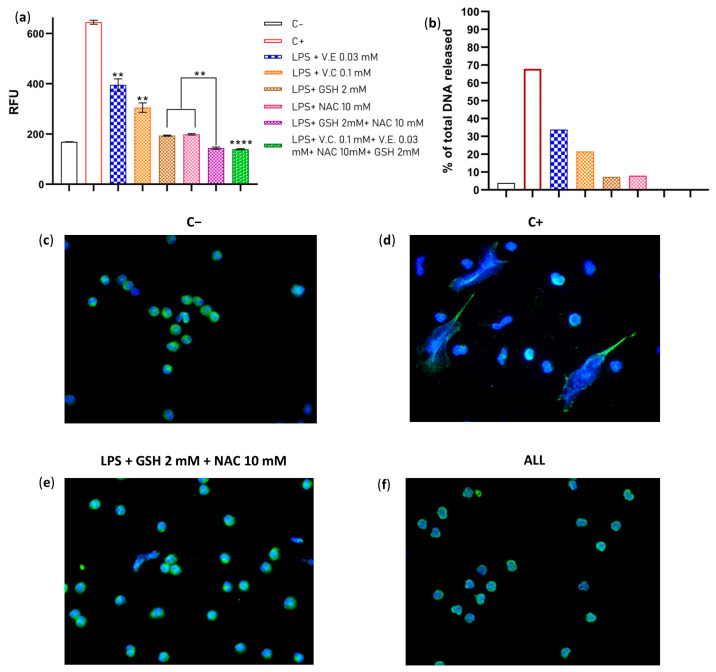
Evaluation of the combined effect of GSH + NAC and ALL antioxidants on NET formation. LPS 10 μg/mL was used as stimuli for the neutrophils. (**a**) Comparison by fluorometry of the effect on NET formation of unsupplemented neutrophils (C−); unsupplemented neutrophil + LPS (C+); neutrophils + vitamin E (0.03 mM) + LPS; neutrophils + vitamin C (0.1 mM) + LPS; neutrophils + GSH (2 mM) + LPS; neutrophils + NAC (10 mM) + LPS; neutrophils + GSH + NAC + LPS, and neutrophils supplemented with ALL antioxidants + LPS. (**b**) Percentage of DNA released. (**c**) Fluorescence microscopy of the negative control (C−). (**d**) Fluorescence microscopy of the positive control (C+) (**e**) Fluorescence microscopy of the neutrophils treated with GSH + NAC + LPS. (**f**) Fluorescence microscopy of the neutrophils treated with ALL + LPS. The experiment had a sample size of 4 subjects for each triplicate. Data were normalized by inverse function and one-way ANOVA, and T3-Dunnet post hoc test was performed. Results are presented as relative fluorescent units (RFU) mean ± S.E. Significant *p*-values: <0.002 (**), <0.0001 (****). Immunofluorescence was performed using DAPI for DNA staining and anti-NE for neutrophil elastase granules, 40× magnification (**c**–**f**), and each image is representative of four independent experiments.

The antioxidant system of the cell works in synergy depending on the redox potential of their molecules and their chemical nature. To assess whether the suppressive effect of antioxidants improves when two or four antioxidants are added simultaneously, we pre-incubated neutrophils with two different antioxidants at previously used concentrations. Vitamins E and C were used at serum levels reported in healthy subjects (0.03 mM and 0.1 mM, respectively), while GSH was used at an intracellular concentration of 2 mM, and NAC was used at the experimental concentration that had the best effect on reducing NETs (10 mM). We found that the combination of GSH + NAC significantly improved its suppressive effect on NET formation when compared to their effects alone. The same effect was observed with ALL antioxidants (Figure 2a,b). Furthermore, fluorescent microscopy confirmed these results, with no NETs found in any sample with supplemented neutrophils (Figure 2c–f). Additionally, we assessed apoptosis and cytotoxicity in the treated neutrophils to confirm that the reduction in NET formation was not due to the death of neutrophils. Our results showed no significant effects on cell viability, suggesting that the observed decrease in NET formation was not a result of neutrophil death (Figures S1 and S2). These results demonstrate that when GSH + NAC and ALL antioxidants are combined, their effect is so strong that it totally abolishes the NET formation. These combinations could improve the TAC of the neutrophils and maintain their redox balance; thus, NETs induced by ROS can be avoided.

### 2.3. The Total Antioxidant Capacity (TAC) of the Neutrophils Is Enhanced When Supplemented with GSH + NAC and ALL Antioxidants

**Figure 3:**
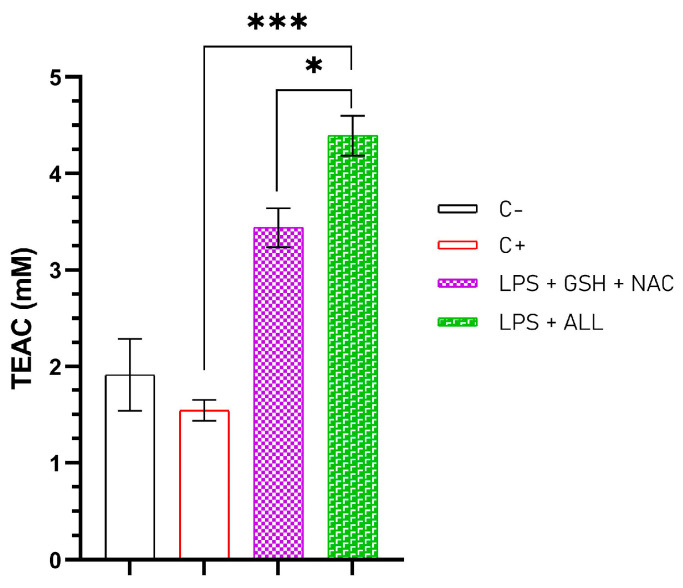
Total antioxidant capacity of neutrophils stimulated with LPS. TEAC concentration in freshly isolated neutrophils (C−), unsupplemented neutrophils stimulated with LPS after 3 h (C+), and pre-incubated neutophils with antioxidants for 1 h and then stimulated with LPS after 3 h (LPS + GSH + NAC and LPS + ALL antioxidants). The experiment had a sample size of 3 subjects for each duplicate. One-way ANOVA and T3-Dunnet post hoc test were performed. Results are presented as mM of Trolox equivalent antioxidant capacity (TEAC) mean ± S.E. Significant *p*-values: ≤0.03 (*), <0.001 (***).

To determine if supplementing antioxidants improves the TAC of neutrophils, we initially assessed the antioxidant capacity of three components: the culture media, autologous serum (AS), and culture media with 2% AS. The culture media (RPMI 1640) exhibited 2.987 mM of Trolox equivalent antioxidant capacity (TEAC), while AS had 3.169 mM TEAC, and the culture media + 2% AS had the highest result with 5.203 mM TEAC (Supplementary Figure S3a). Furthermore, the culture media showed a significant improvement after antioxidant supplementation, particularly in those treated with GSH + NAC and ALL antioxidants (Supplementary Figure S3b). Upon determining the TAC of the culture media used in our experiments, we assessed the TAC of neutrophils incubated with antioxidants and stimulated with LPS for 3 h. Freshly isolated neutrophils exhibited a TEAC of 1.653 mM, which increased to 1.912 mM TEAC after one hour of pre-incubation in culture media with AS; however, after 3 h of stimulation with LPS, the TEAC values fell to 1.543. Moreover, pre-incubated neutrophils with GSH + NAC and ALL antioxidants demonstrated TEAC values of 3.437 and 4.391 mM, respectively, despite being stimulated with LPS for 3 h (Figure 3). These results indicate that in vitro antioxidant supplementation of neutrophils enhances their antioxidant capacity, mainly in those that contain GSH + NAC, as well as ALL antioxidants. However, it is necessary to prove if the redox state of the neutrophils is improved with the use of antioxidants.

### 2.4. The Redox State (GSH/GSSG) of the Neutrophils Is Improved When Supplemented with GSH + NAC or ALL Antioxidants

**Figure 4:**
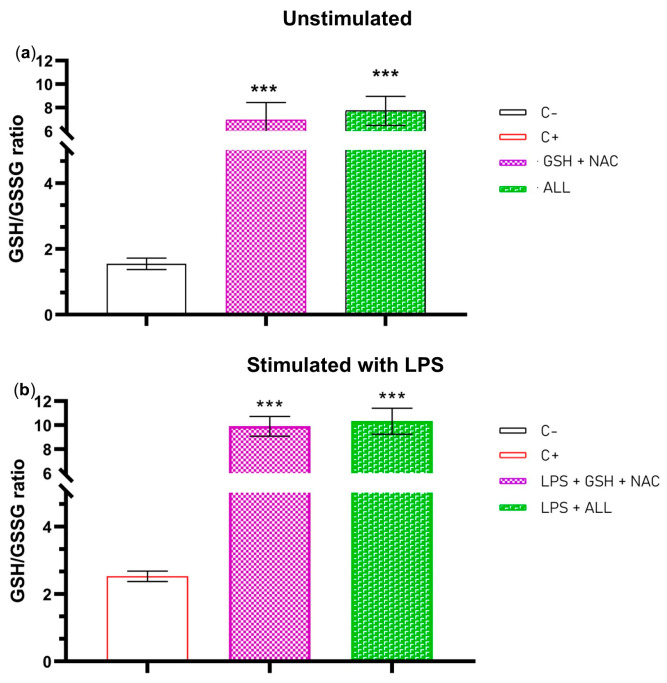
Redox state of the neutrophils. (**a**) The GSH/GSSG ratio was evaluated in unsupplemented neutrophils (C−) and in supplemented neutrophils with GSH + NAC and ALL antioxidants for 1 h. (**b**) GSH/GSSG ratio in unsupplemented (C+) and supplemented neutrophils stimulated with LPS after 3 h. The experiment had a sample size of 3 subjects for each duplicate. One-way ANOVA and T3-Dunnet post hoc test were performed. Results are presented as GSH/GSSG ratio mean ± S.E. Significant *p*-values: <0.001 (***).

We evaluated the redox state of neutrophils by measuring the reduced glutathione/oxidized glutathione (GSH/GSSG) ratio before (freshly isolated neutrophils) and after pre-incubation with antioxidants. Freshly isolated neutrophils exhibited a ratio of 1.547, which significantly improved to 6.9541 with GSH + NAC and 7.731 with ALL antioxidants (Figure 4a). Furthermore, when neutrophils were stimulated with LPS, the redox state increased slightly to 2.526 in the group without antioxidants, but it significantly increased to 9.9138 and 10.338 in those supplemented with GSH + NAC and ALL antioxidants, respectively (Figure 4b). These results demonstrate that antioxidant combinations, such as GSH + NAC and ALL antioxidants, can increase the GSH/GSSG ratio and improve the cell’s redox state. Although the mechanism by which neutrophils increase their GSH/GSSG ratio is unknown, this increase may be a protective response against lipid peroxidation.

### 2.5. The Amount of ROS Is Sustained at Basal Levels in Supplemented Neutrophils with GSH + NAC and ALL Antioxidants

**Figure 5:**
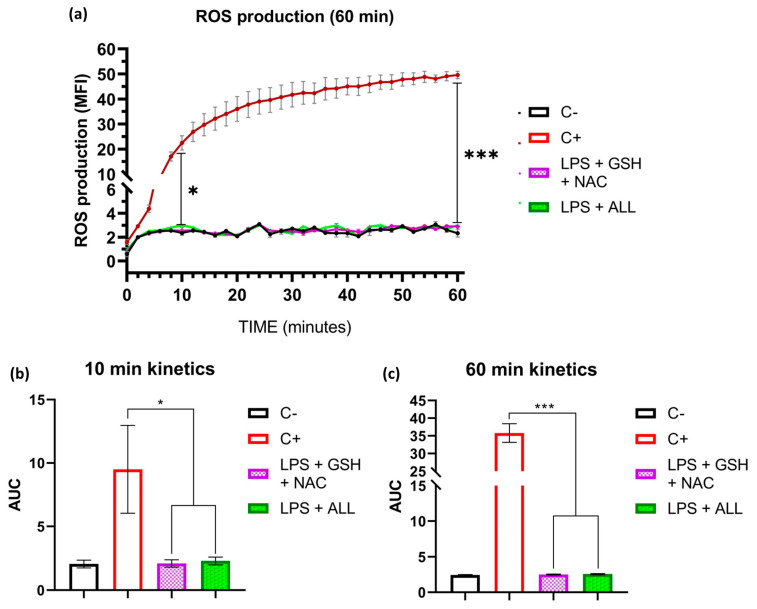
Antioxidants reduce ROS production. The kinetics of ROS production by neutrophils were analyzed to investigate the scavenging process under stimulation by LPS. The effect of GSH + NAC and ALL antioxidants on ROS production was determined. ROS quantification was measured using median fluorescence intensity (MFI) and represented by the area under the curve (AUC). Freshly isolated neutrophils (1.5 × 10^5^) were prepared in RPMI 1640 (pH 7.3) + 2% AS media and incubated with the DCFDA probe. The kinetic curve was performed for 60 min, with measurements taken every 2 min. The positive control and antioxidant groups (GSH + NAC and ALL) were stimulated with 10 µg/mL of LPS. (**a**) ROS production kinetics. Statistical differences were found after 10 min between the C+ and antioxidant groups. The differences were maintained for 60 min. (**b**) AUC of ROS production at the first 10 min. (**c**) AUC of ROS production after 60 min. The sample size was 4 subjects for each triplicate. Statistical analysis was performed using one-way ANOVA and T3-Dunnet post hoc test to analyze the AUC values. Results are presented as mean ± S.E. of MFI. Significant *p*-values: <0.03 (*), <0.001 (***).

To evaluate ROS scavenging by antioxidants, we first measured the production of ROS with the non-specific probe 2′,7′-dichlorodihydrofluorescein in its diacetate form (DCFH-DA) for 180 min. In the first 60 min, we measured ROS every 2 min, and after that, we performed measurements at 90, 120, and 180 min (Supplementary Figure S4). We found that in the first 60 min, the negative control (neutrophils in RPMI 1640 + 2% AS) and the antioxidant-treated neutrophils with GSH + NAC and ALL presented similar values of median fluorescence intensity (MFI), even though these last mentioned were stimulated with LPS, whereas the neutrophils without antioxidant supplementation that were stimulated with LPS increased ROS production significantly and reached an average of 49.58 MFI (Figure 5a). When comparing the area under the curve (AUC) of the ROS production at 10 and 60 min, our findings showed that the combinations of GSH + NAC and ALL antioxidants significantly maintained the ROS levels near basal values throughout both evaluated time intervals (Figure 5b,c). These results demonstrate that both combinations were effective in reducing ROS production from the beginning of LPS stimulation and maintained their suppressive effect until the end of the experiment.

### 2.6. The Combinations of GSH + NAC and ALL Antioxidants Decrease Nitrite + Nitrate Concentration in LPS-Stimulated Neutrophils

**Figure 6:**
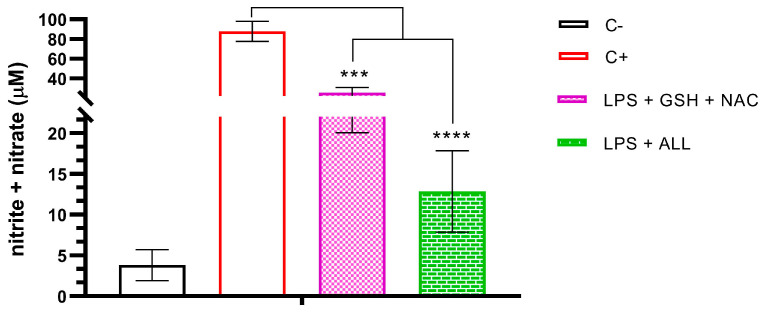
Nitrite/nitrate production in neutrophils supplemented with antioxidants and stimulated with LPS. Assessment of nitrite + nitrate production was performed in unsupplemented neutrophils (C−), unsupplemented neutrophils stimulated with LPS, and supplemented neutrophils with the antioxidant combinations of GSH + NAC or ALL stimulated with LPS. Measurements were taken after 3 h of stimulation with LPS. The sample size was 3 subjects for each triplicate. Statistical analysis was performed using one-way ANOVA and T3-Dunnet post hoc test. Results are presented as mean ± S.E. of nitrite + nitrate concentration (µM). Significant *p*-values: <0.001 (***), <0.0001 (****).

To evaluate the effect of antioxidants on nitrite + nitrate levels, we first measured their concentration in freshly isolated neutrophils. Next, we measured the levels in neutrophils pre-treated with GSH + NAC and ALL antioxidants, which were then stimulated with LPS. We found that the nitrite + nitrate levels were 3.815 µM in freshly isolated neutrophils, and this level increased to 87.807 µM when the neutrophils were stimulated with LPS. However, when the neutrophils were pre-incubated with GSH + NAC, the effect of LPS on nitrite + nitrate concentration was reduced to 25.397 µM. Moreover, the effect was even stronger with ALL antioxidants, which reduced the nitrite + nitrate levels to 12.852 µM (Figure 6).

### 2.7. The Neutrophils Supplemented with Antioxidants Maintain Basal Levels of MDA and Prevent Lipid Peroxidation When Stimulated with LPS

**Figure 7:**
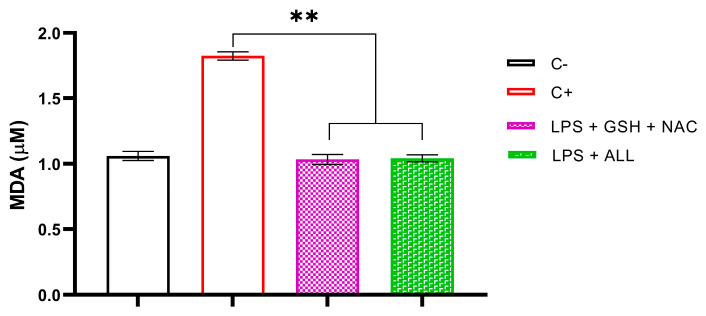
Effect of the combinations GSH + NAC and ALL antioxidants on neutrophil’s lipid peroxidation. The assessment of lipid peroxidation was carried out in unsupplemented neutrophils (C−), unsupplemented neutrophils stimulated with LPS after 3 h (C+), and supplemented neutrophils with antioxidants (GSH + NAC and ALL) stimulated with LPS after 3 h. The sample size was 3 subjects for each duplicate. Statistical analysis was performed using one-way ANOVA and T3-Dunnet post hoc test. Results are presented as mean ± S.E. of MDA concentration (µM). Significant *p*-values: <0.002 (**).

To assess the impact of antioxidant combinations on lipid peroxidation, we measured the level of MDA in neutrophils. Initially, we determined the baseline levels of MDA in freshly isolated neutrophils, which were found to be 1.06 µM. Subsequently, we measured the MDA levels after stimulation with LPS, which resulted in a significant increase up to 1.8257 µM. However, we observed that neutrophils supplemented with antioxidants maintained normal levels of MDA even after the addition of LPS. Specifically, GSH + NAC-treated neutrophils exhibited MDA levels of 1.034 µM, while ALL antioxidants resulted in MDA levels of 1.042 µM. In all cases, MDA levels remained significantly unaltered until the end of the experiment (Figure 7). These findings demonstrate that antioxidant supplementation prevents the lipid peroxidation of membrane complexes in neutrophils, possibly through ROS scavenging by antioxidants.

## 3. Discussion

ROS has been shown to play a crucial role in the process of NET formation. As a result, researchers have studied antioxidants over the past decade to control them both in vivo and in vitro. The vast majority of the available studies were performed with the chemical stimulus of PMA, a synthetic molecule that directly activates the NADPH complex without involving any receptor [40]. This feature makes PMA less representative of physiological events. In the present work, we used LPS from *E. coli* in our experiments since this molecule is present in human blood in normal and pathological conditions. Furthermore, this molecule is present in the outer membrane of Gram-negative bacteria and is capable of interacting with neutrophil receptors to induce ROS production and undergo NETosis [41,42].

Recent studies have investigated the effect of vitamin C on NET formation. In our investigation, we found that vitamin C can reduce LPS-induced NETs in a dose-dependent manner from 0.003 mM to 3 mM. This is consistent with Kirchner and Mohammed’s previous findings when they used concentrations of 2 mM and 3 mM, respectively. Interestingly, we found that even at concentrations of vitamin C that were considered deficient (0.003 mM) and insufficient (0.03 mM) in humans, there was a decrease in NET formation, and the effect became stronger with the concentration of serum levels (0.1 mM) and intracellular levels reported in neutrophils (1.5 mM). This contrasts with Kirchner and Mohammed’s findings, who did not observe any reduction using lower concentrations (0.2 and 0.3 mM, respectively). The differences in results may be due to the use of different NET inducers: PMA in Kirchner and Mohammed’s studies and LPS from *E. coli* in our study. Additionally, it should be mentioned that we added the antioxidant to the media, which means that vitamin C could be available for neutrophils if needed. This is important because neutrophils can reach intracellular concentrations of up to 1.5 mM of vitamin C through the SVCT2 transporter [43], and when activated with PMA, concentrations can increase by two orders of magnitude due to the transport of its oxidized form via GLUT3 [44]. Our results reinforce previous findings of vitamin C on NET formation in vitro and demonstrate that the availability of ascorbic acid, even at low levels, is fundamental in the context of NET formation, and underscore the importance of vitamin C in neutrophil function due to its high solubility, efficient transport properties within the cell, and its ability to interact with vitamin E to maintain cell membrane integrity.

Vitamin E is present in all cell membrane complexes and has been shown to protect against lipid peroxidation by indirectly reacting with ROS [24]. In our study, we found that concentrations of vitamin E ranging from 0.03 to 1 mM reduced NET formation in neutrophils stimulated with LPS in a dose-dependent manner. This contrasts with Kirchner et al. (2013), who did not observe any reduction of NET formation in human neutrophils supplemented with 0.05 mM of α-tocopherol. This discrepancy may be due to the difference in vitamin E dosages and the use of PMA as a stimulus. Another study carried out by Vorobjeva et al. reported similar results to ours using a soluble analog of vitamin E called Trolox at concentrations of 0.2, 0.5, and 1 mM [45]. They found a dose-dependent effect on reducing NET formation. However, Trolox is localized in the soluble portions of the cell, making this molecule difficult to compare with the physiological role of α-tocopherol.

Plasma levels of GSH in healthy subjects are reported to be 800 µM, while most cells present higher intracellular concentrations ranging from 2 to 10 mM. Our study found that supplementing neutrophils with 0.8 mM and 2 mM of GSH resulted in a dramatic reduction of NETs, with an average of 50% and 55% fewer NETs, respectively. The effect was slightly better than that with the highest concentrations of vitamins C and E, possibly due to the higher molar concentration of GSH and its electronegativity compared to that of vitamins E and C. Our findings emphasize the significance of GSH in controlling NETs and prompt further investigation into how neutrophils utilize GSH, given the difficulties of transporting it inside the cell. Further research is necessary to better comprehend the mechanisms underlying the GSH effects on NET formation and its potential therapeutic applications.

NAC is a commonly used medication for treating acetaminophen toxicity and has been studied for its antioxidant properties in treating free radical damage [46]. Cysteine is known to be the rate-limiting step in the synthesis of GSH, and NAC provides L-cysteine to support GSH maintenance [47]. Our experiments showed that NAC can suppress NET formation when used at high concentrations (10 mM), which is consistent with Kirchner’s findings that 10 mM effectively reduced NET formation in vitro. However, we did not observe a significant effect at the concentration of 5 mM, which contrasts with the results of Kirchner, who used the same concentration and found a significant reduction. This discrepancy may be due to differences in the stimuli used in Kirchner’s study (PMA) and that used in ours (LPS). Additionally, it must be considered that once NAC is deacetylated, cysteine is rapidly oxidized to cystine, which may limit its antioxidant effect at low concentrations [48]. Further research is needed to better understand the effect of NAC on NET formation induced by a natural stimulus.

As previously mentioned, some antioxidants work together inside the cell to enhance antioxidant capacity. For example, vitamin E, vitamin C, and GSH interact to improve antioxidant function. Other molecules, such as NAC, provide the necessary components for the synthesis of GSH. Our study tested two combinations of antioxidants for the first time: (1) GSH (2 mM) + NAC (10 mM) and (2) vitamin E (0.03 mM) + vitamin C (0.1 mM) + GSH (2 mM) + NAC (10 mM). We found that the combination of GSH + NAC had a suppressive effect on NET formation that reached basal levels. Furthermore, when we tested ALL antioxidants, we observed an improvement in the suppressive effect on NET formation when compared with the combination of GSH + NAC, although this difference was not statistically significant. It is possible that the combination of ALL may have a differential effect on other parameters related to oxidative stress. Moreover, it is important to note that the amounts of vitamin E and vitamin C used were insignificant compared to the concentrations of NAC and GSH. Therefore, this may be the reason why we did not observe a significant effect when comparing both combinations. In addition to our main findings, we conducted further assessments to evaluate the effect of supplemented antioxidants on neutrophil viability. Specifically, we performed determinations of apoptosis/necrosis and cytotoxicity using flow cytometry and colorimetric techniques, respectively. Our results revealed no significant differences in viability between the negative controls and the neutrophils supplemented with antioxidants in both experiments (Figure S1). These findings indicate that the supplementation of antioxidants does not have an adverse effect on neutrophil viability. Our experiments demonstrate for the first time that these molecules can interact with each other, improving the neutrophil capacity to avoid NET formation when stimulated with LPS in vitro.

The TAC of neutrophils has been studied in various contexts, including exercise, infection, and disease. For example, studies have shown that antioxidant supplementation can enhance the activity of antioxidant enzymes in neutrophils, while oxidative stress can impair neutrophil function [49]. Researchers have studied the effects of antioxidants, including vitamin E, vitamin C, and NAC, on NET formation. However, the determination of the TAC in this context has yet to be fully understood. In our experiments, we observed an increase in TAC when comparing freshly isolated neutrophils to those that were supplemented with antioxidants. The TAC of freshly isolated neutrophils doubled in value compared with neutrophils supplemented with GSH + NAC and ALL the antioxidants. In both cases of supplemented neutrophils, the improvement observed was twice as high as the basal levels. We hypothesized that increasing the amount of antioxidants inside the neutrophil cells could reduce NET formation by improving its TAC. These findings highlight the significance of comprehending the TAC of neutrophils and the role of antioxidant availability in preserving their function and safeguarding against oxidative damage. However, further studies are necessary to clarify the mechanisms involved in how this increase in TAC is achieved.

The ratio of GSH to GSSG is an essential indicator of cellular redox balance in various cell types, including immune cells. The GSH/GSSG ratio is used as an indicator of cellular oxidative stress. Oxidative perturbations can cause a redistribution of GSH and GSSG, resulting in a shift in the GSH/GSSG ratio, which reflects the oxidized redox status within a cell [47,48]. Our study revealed that freshly isolated neutrophils had a GSH/GSSG ratio of 1.547. However, when supplemented with GSH + NAC or ALL antioxidants, the ratio significantly improved to 6.954 and 7.731, respectively. Furthermore, when we stimulated the supplemented neutrophils with LPS for 3 h, their GSH/GSSG ratio increased to 9.913 in the group with GSH + NAC and 10.333 in the group with ALL antioxidants. No reports have been found regarding GSH/GSSG levels in neutrophils. However, previous studies have reported that the GSH/GSSG ratio in the monocytes and T cells of healthy children ranges from 12 to 25 and from 10 to 24, respectively [50]. Our findings show similar values to these previous reports, although it must be considered that our measurements were performed in neutrophils from healthy young adults that were seeded in cell culture media (RPMI 1640) + 2% AS supplemented with a mixture of antioxidants (GSH + NAC and ALL) and stimulated with LPS. The concentration of the GSH/GSSG ratio may vary depending on several factors, including the subjects involved, the specific media used, and the interactions among the molecules present. Regarding the increments in the redox potential after stimulation with LPS, this could be because of the antioxidant-enriched environment, the interaction of these antioxidants, and the activation of antioxidant response elements (AREs) after LPS stimulation through Nrf2 [51] that promotes GSH synthesis [52], although these assumptions should be proven with further experiments. While most studies on the GSH/GSSG determination have focused on brain cells or tissues, just a few studies have been conducted on immune cells. This is the first time that GSH/GSSG ratio is measured in neutrophil cultures and provides new information about its function in NET formation. However, further experiments are needed to describe the kinetics of the redox potential maintenance in supplemented neutrophils.

ROS and NET formation have been the focus of recent investigations in various diseases; however, the biological regulation during this process remains to be elucidated. Our experiments showed that the levels of ROS increased significantly in the first 10 min in the positive control and reached another peak at 60 min. The neutrophils that were pre-incubated with the combinations of antioxidants GSH + NAC and ALL showed values similar to those observed in the negative control, despite being stimulated with LPS. In 2012, Kirchner et al. [9] reported that the formation of NETs depends on the generation of ROS, and in 2013, they conducted a study on the effect of various antioxidants on ROS production in NET formation. They found that neutrophils supplemented with vitamin C at concentrations of 0.2 and 2 mM showed a significant reduction in ROS production [32]. Our findings provide novel information on the antioxidant effects of GSH + NAC and ALL antioxidants on ROS production. Both combinations could reduce ROS levels to near basal levels, emphasizing the importance of antioxidant supplementation for neutrophils to prevent oxidative stress. However, further research is needed to elucidate the type of ROS that are being scavenged.

It has been described that RNS plays an important role in NET formation; however, there is limited information available on this topic. We measured, for the first time, nitrite (NO_2_^−^) + nitrate (NO_3_^−^) levels as RNS indicators in supplemented neutrophils. In our results, we found basal levels of 3.81 µM of NO_2_^−^ + NO_3_^−^, and this concentration increased significantly to 87.80 µM after LPS stimulation. We also found that the combination of GSH + NAC and ALL antioxidants significantly reduced nitrite + nitrate levels to 25.39 and 12.85 µM, respectively. The basal values that we found are higher than previously reported by Singh et al. (2.8–3 µM/10^7^ cells) [53]; however, the increase after stimulation is consistent with previous studies that have assessed NO and ONOO^−^ [14,15]. It has been described that NO undergoes various reactions in biological fluids, resulting in the production of nitrite (NO_2_^−^) and nitrate (NO_3_^−^); however, it must be considered that nitrite/nitrate levels do not reflect the amount of NO or ONOO^−^. Here, we describe that these final products augment during NET formation but can be reduced through the action of antioxidant combinations such as GSH + NAC and ALL antioxidants. Further investigations are necessary to comprehend the role of RNS in neutrophils, which can provide valuable insights into the mechanisms of NET formation.

MDA is a widely used biomarker of oxidative stress and lipid peroxidation. Several studies have investigated the role of MDA in neutrophils and its association with various diseases. We measured the MDA levels in freshly isolated neutrophils in our experiments and observed a significant increase after LPS stimulation from 1.06 to 1.82 µM of MDA. Our findings also showed that combining GSH + NAC and ALL antioxidants significantly prevented the increment of MDA and maintained basal levels (1.03 and 1.04 µM of MDA, respectively). MDA levels have been reported in various cell culture assays, including cardiomyocytes (4 µM) [54], neurons (0.4 nmol/mg) [55], and serum (0.2 µM) [56], among others. However, MDA levels in neutrophil cultures have not yet been determined. These describe, for the first time, the MDA levels in cultured neutrophils and compare their levels after stimulation with LPS. Furthermore, we demonstrate that the antioxidant combinations used can avoid lipid peroxidation in neutrophils. However, it must be considered that MDA is also formed from other compounds such as deoxyribose, sodium benzoate, and flavone C glucosides, among others. Therefore, further experiments are needed to elucidate the implications of lipid peroxidation on NET formation.

## 4. Materials and Methods

### 4.1. Isolation of Neutrophils

Neutrophils were obtained from a total of 8 healthy donors in a fasting state by venous puncture in EDTA-containing tubes (EDTA K2, BD Vacutainer^®^). A double gradient was formed in a 15 mL tube with the blood and the reagents Histopaque^®^1119 and 1077 (Sigma Aldrich, St. Louis, MO, USA). Then, the tube was centrifuged at 2200 rpm for 30 min at room temperature for phase separation of the blood components and the recovery of polymorphonuclear (PMNs) cells. The PMNs were resuspended in 1 mL of 0.9% sodium chloride. A viability assay was performed in a Neubauer chamber with trypan blue (1:4 PMNs/trypan blue). Viability values higher than 95% were required for the experiments.

### 4.2. Culture Media Preparation

The negative and positive control media were prepared using RPMI 1640 without phenol red, buffered with 2% sodium bicarbonate to maintain a physiological pH of 7.4 (±0.1), and supplemented with 2% autologous serum (AS). To prepare the antioxidant-supplemented media, we followed the same procedure as the controls but with the addition of freshly prepared antioxidants at the required concentrations. For media supplemented with vitamin C, we prepared a solution using L-ascorbic acid from Sigma Aldrich (Cat. No. A4544) in RPMI 1640 without phenol red. The final concentrations in the media were 0.003, 0.03, 0.1, 1.5, and 3 mM. The media supplemented with vitamin E was prepared using α-tocopherol from Sigma Aldrich (Cat. No. 258024), following the manufacturer’s instructions. The final concentrations were 0.03, 0.3, and 1 mM. For GSH supplementation, L-glutathione reduced from Sigma Aldrich (Cat. No. G4251) was prepared in ddH_2_O water for 10 min before adding it to the media. The final concentrations were 0.8 and 2 mM. The GSH solution was always protected from light. The NAC-supplemented media was prepared by diluting N-acetyl-L-cysteine from Sigma Aldrich (Cat. No. A7250) in ddH_2_O water and adding it to the media at final concentrations of 5 and 10 mM. The combination of GSH + NAC was prepared by adding L-glutathione reduced at a final concentration of 2 mM and N-acetyl-L-cysteine at a final concentration of 10 mM. The combination of all antioxidants (ALL) was prepared by adding L-ascorbic acid at a final concentration of 0.1 mM, (±)-α-tocopherol at a final concentration of 0.03 mM, L-glutathione reduced at a final concentration of 2 mM, and N-acetyl-L-cysteine at a final concentration of 10 mM.

### 4.3. NETosis Assay

In total, 2.5 × 10^5^ cells per well were seeded in a 24 multi-dish plate (Cat. No. 143982, Nunclon). The negative and positive controls and the antioxidant-supplemented samples were seeded with their corresponding media (described in Section 4.2) and incubated for 60 min. The positive control and the antioxidant samples were stimulated with 10 µg/mL of LPS from *E. coli* (Cat. No. L3129, Sigma Aldrich) and incubated for 180 min at 37 °C and 0.5% of CO_2_.

### 4.4. NETs Microscopy

After inducing NETosis in a 24-well plate (Cat. No. 3524, Costar, Washington, DC, USA), previously treated with poli-L-lysine (P4707, Sigma Aldrich), immunofluorescent imaging was performed. The cells were fixed with 4% paraformaldehyde (158127, Sigma Aldrich) for 10 min. Following fixation, the samples were washed twice with cold PBS 1× and then stained with DAPI at a concentration of 2 µg/mL (32,670, Sigma Aldrich) overnight at 4 °C. For elastase staining, a primary antibody against elastase (1:500) was used. Neutrophils, which were previously stained with DAPI, were washed twice in the dark and then incubated with the primary antibody for 60 min at room temperature, protected from light. Subsequently, a secondary antibody, anti-IgG conjugated with Alexa fluor 594 (A11012, Life technologies, Carlsbad, CA, USA), was added and incubated for 60 min. Images were captured at 40× magnification using a fluorescence microscope (Carl ZEISS Z1, Berlin, Germany). Representative fields from four different subjects were considered for analysis.

### 4.5. NETs Fluorometry

After inducing NETosis in a 24-well plate (Cat. No. 3524, Costar), the plate was centrifuged at 2000 rpm for 10 min, and then a volume of 750 μL of supernatant was discarded. Subsequently, 1 µL of DNase I (AMPD1, Sigma Aldrich) was added to each well, and the plate was incubated for 30 min at 37 °C. Following the incubation, the plate was centrifuged again at 2000 rpm for 10 min. From each well, 300 μL of the supernatant was transferred to a dark 96-well plate. Next, 5 mM of SYTOX^®^ Green (Cat. No. S7020, Thermo Fisher Scientific, Carlsbad, CA, USA) was added to each well and incubated at room temperature for 15 min. Fluorescence readings were performed using the Biotek Synergy HTX multimode reader from Agilent with excitation at 485 nm and emission at 527 nm filters. The results were quantified in relative fluorescent units (RFU). The percentage of DNA released was determined by comparing the RFU values of the samples to the RFU values obtained from 2.5 × 10^5^ lysed neutrophils with 10% triton X-100 (Cat. No. 85111, Thermo Fisher), which was considered as 100% of the total DNA.

### 4.6. Dead Cell Assay by Flow Cytometry

To assess the markers of dead cells for apoptosis and necrosis, we employed the Dead Cell Apoptosis Kit with Annexin V FITC and Propidium Iodide for flow cytometry from Invitrogen (Cat. No. V13242). Neutrophils were seeded at a concentration of 2 × 10^5^ cells per milliliter in conical tubes. The control and antioxidant-supplemented neutrophils (GSH + NAC and ALL) were pre-incubated for 60 min with their respective media (see material and methods 4.2). After the pre-incubation period, the cells were washed with PBS 1× and resuspended in 0.5 mL of 1× annexin-binding buffer (provided in the kit). Then, 5 µL of FITC Annexin V and 1 µL of propidium iodide (PI) working solution were added for each 100 µL of solution. The samples were incubated at room temperature in the dark for 15 min. Following the incubation, 400 µL of 1× annexin-binding buffer was added. The samples were acquired using the Attune Acoustic Focusing Cytometer (Life Technologies, Carlsbad, CA, USA), with 20,000 events per sample being acquired. The filters used were BL1-A (FITC) and BL2-A (PI). The analysis was performed using FlowJo™ v. 10.9.0 software (BD Biosciences).

### 4.7. Cytotoxicity Assay

The cytotoxicity assay was conducted using the CyQUANT™ MTT Cell Proliferation Assay Kit from Invitrogen (Cat. No. V13154). Neutrophils were seeded at a concentration of 2 × 10^5^ cells per milliliter in conical tubes. The control and antioxidant-supplemented neutrophils (GSH + NAC and ALL) were pre-incubated for 60 min with their respective media (see materials and methods 4.2). After the pre-incubation period, the neutrophils were washed twice with PBS 1× to remove the media. Then, 100 µL of freshly prepared RPMI 1640 media alone was added to each well of a Nunc Flat-bottom microplate from Thermo Fisher (Cat. No. 168055), with a concentration of 2 × 10^5^ cells per well. Subsequently, 10 µL of the CyQUANT™ MTT reagent was added to each well. The negative control of RPMI 1640 + MTT was included, as indicated by the manufacturer’s protocol. The plate was incubated for 3 h at 37 °C. After the incubation period, the plate was centrifuged for 10 min at 4 °C and 2000 rpm. Then, 85 µL of the total volume was carefully removed, and 50 µL of dimethyl sulfoxide (DMSO) was added to each well. The plate was incubated for 15 min, and each well was resuspended prior to reading. Absorbance readings were performed using a microplate reader (ALLSHENG) at 540 nm.

### 4.8. Total Antioxidant Capacity Assay

To evaluate the antioxidant effect in both medium and neutrophils, we utilized the Total Antioxidant Capacity Assay Kit from Abcam (Cat. No. ab65329, Cambridge, UK) following the manufacturer’s protocol without the protein mask. The TAC was assessed in the culture media components: RPMI 1640 alone, AS alone, and RPMI 1640 + AS, following the manufacturer’s instructions for liquid samples. For the evaluation of TAC in neutrophils, we seeded 1 × 10^6^ neutrophils and followed the same protocol described in the section of NETosis assay for the controls and the combination of antioxidants GSH + NAC and ALL. After inducing NETosis, in all cases, the cell suspensions were washed twice with cold PBS 1× to remove the media. Subsequently, the samples were resuspended in 100 µL of ddH_2_O containing 0.05% Triton X-100 (Cat. No. 85111, Thermo Fisher). The resulting homogenate was then incubated on ice for 10 min. After incubation, the samples were centrifuged at top speed for 5 min at 4 °C. The supernatant was collected, and the assessment was carried out according to the instructions provided in the kit. The output of the TAC assay was measured using a microplate reader (ALLSHENG, Hangzhou, China) at 570 nm.

### 4.9. Redox State of the Cell (GSH/GSSG)

The redox state of neutrophils was performed using the GSH/GSSG Ratio Detection Assay Kit from Abcam (Cat. No. ab138881). For each experimental condition, 5 × 10^5^ neutrophils per milliliter were seeded in 1.5 mL tubes, following the same protocol described in the NETosis assay section for the controls and the supplemented neutrophils with GSH + NAC and ALL antioxidants. Two measurements were taken: one after 60 min of antioxidant pre-incubation and the other after 180 min of LPS stimulation. Similar to the TAC assay, all cell suspensions were washed twice with cold PBS 1× to remove the media. The cells were then sonicated with 5 cycles of 5 s with intervals of 5 s off. Subsequently, the samples were centrifugated for 15 min at 4 °C at top speed. The supernatant was collected, and the analysis was performed according to the manufacturer’s protocol provided with the assay kit. Fluorescence readings were conducted using the Agilent Biotek Synergy HTX multimode reader with excitation at 490 nm and emission at 520 nm filters.

### 4.10. ROS Production Assessment

The neutrophil’s ROS production during NET formation was assessed using the DCFDA/H2DCFDA—Cellular ROS Assay Kit from Abcam (Cat. No. ab113851). For each experimental condition, we incubated 5 × 10^5^ neutrophils per milliliter with the DCFDA probe for 30 min. Next, the neutrophils were washed twice with cold PBS 1×, and the samples were pre-incubated for 60 min with their respective media as described in the media preparation section for controls and antioxidant combinations (GSH + NAC and ALL). After the pre-incubation, the samples were washed twice with cold PBS 1×, and the media was replaced with freshly prepared RPMI 1640. Subsequently, 1.5 × 10^5^ neutrophils per well were seeded in a black opaque 96-well plate. The experiment was performed in triplicates for each condition to ensure reproducibility. ROS production was monitored at 2 min intervals during the initial 60 min, and additional measurements were taken at 90, 120, and 180 min to capture the dynamic changes over time. Fluorescence readings were acquired using the Agilent Biotek Synergy HTX multimode reader with excitation at 485 nm and emission at 535 nm filters.

### 4.11. Nitrite + Nitrate Production Assay

To evaluate nitrite + nitrate production in neutrophils, we used the Nitrate/nitrite Colorimetric Assay Kit from Cayman (Cat. No. 780001, Ann Arbor, USA). Neutrophils were seeded at a concentration of 1 × 10^6^ cells per milliliter in 1.5 mL conical tubes. The samples were pre-incubated for 60 min with their respective media as described in the media preparation section for controls and the antioxidant combinations (GSH + NAC and ALL). After pre-incubation, the cell suspensions were washed twice with cold PBS 1×, and the protocol described for NETosis assay was conducted. Subsequently, the cell suspensions were sonicated with 5 cycles of 5 s with intervals of 5 s off. The cells were centrifugated at 14,000 rpm for 30 min, and the supernatant was collected. The nitrite/nitrate assay was conducted using a volume of 80 µL of the processed samples, following the manufacturer’s protocol provided with the kit. The absorbance was measured at 550 nm using a microplate reader (ALLSHENG).

### 4.12. MDA Determination

The generation of MDA by stimulated and unstimulated neutrophils was measured using the QuantiChrom^TM^ TBARS Assay Kit (Cat. No. DTBA-100, Bioassay Systems, Hayward, USA) following the manufacturer’s instructions. Neutrophils were seeded at a concentration of 5 × 10^5^ cells per milliliter in 1.5 mL conical tubes, and the procedure described in the NETosis assay section was followed for the controls and antioxidants combinations (GSH + NAC and ALL). MDA levels on freshly isolated neutrophils were determined (negative control). After NETosis assay, the cell suspensions were sonicated with 5 cycles of 5 s with intervals of 5 s off. Subsequently, the samples were incubated for 5 min with trichloroacetic acid (TCA) in a 2:1 proportion (TCA: cell lysate). Following incubation, the samples were centrifugated for 5 min at 14,000 rpm, and 200 µL of the supernatant was transferred to 0.5 mL conical tubes. The fluorometric assay procedure provided with the kit was followed to measure MDA levels. Fluorescence readings were performed using the Agilent Biotek Synergy HTX multimode reader with excitation at 530 nm and emission at 550 nm filters.

### 4.13. Statistics

Data analysis was conducted using the statistical package SPSS (IBM) and GraphPad Prism 8. One-way ANOVA was performed for all experiments, and in certain cases, data normalization (inverse function) was applied as indicated on the corresponding figures. T3-Dunnett post hoc tests were performed, and statistical significance was determined based on the following *p*-values: ≤0.03 (*), ≤0.002 (**), ≤0.001 (***), and ≤0.0001 (****). The specific comparison groups were defined in each respective results section.

## 5. Conclusions

It has been demonstrated that LPS is found in human blood in health and disease conditions. Therefore, it is important to conduct investigations with this type of inductor to properly describe physiological effects. In our investigation, we have demonstrated that vitamin C is highly effective in reducing LPS-induced NETs, even at low doses that are considered insufficient or deficient. We also demonstrated that vitamin E and GSH are effective at doses reported in human serum, with a stronger effect at higher concentrations. By combining antioxidants, such as GSH + NAC and ALL, the reduction of NETs is more effective compared to using antioxidants alone, and these combinations positively affect various parameters, including TAC, redox state of the cell, ROS and RNS production, and lipid peroxidation. The findings of our study provide new information about neutrophil biology and raise several important questions regarding the role of antioxidant molecules in these cells. Our results suggest a potential link between antioxidants and neutrophil function, mainly in controlling NET formation. However, further investigations are necessary to fully understand the underlying molecular mechanisms involved in the antioxidant function, including the role of enzymes like MPO, antioxidant enzymes such as superoxide dismutase (SOD) nitric oxide synthase (NOS), catalase, glutathione S-transferase (GST), and glutathione peroxidase (GPx), as well as to evaluate whether supplemented neutrophils can effectively eliminate bacteria or fungi. By delving deeper into these mechanisms, we will be able to gain a more comprehensive understanding of how antioxidants impact neutrophil biology and potentially develop novel therapeutic strategies. Additionally, future studies could explore the potential interactions between antioxidants and other cellular processes, such as inflammation and immune response, to provide a more holistic view of their role in neutrophil function. Overall, these results highlight the need for continued research in this area to unravel the complexities of antioxidant-mediated processes in neutrophils and their implications for human health.

## Figures and Tables

**Table 1:** Properties of vitamins E and C, GSH, and NAC, and their reported concentrations.

Molecule	^1^ ORP (mV)	Concentration	Chemical Nature	References
Vitamin E	+0.37	0.03 mM (serum)	Hydrophobic	[28,29]
		0.13 mM (adrenals)		
		0.15 mM (adipocytes)		
Vitamin C	+0.08	0.003 mM (insufficient)	Hydrophilic	[28,30,31]
		0.03 mM (deficient)		
		0.1 mM (serum)		
		1.5 mM (inside neutrophil)		
		3 mM (pharmacological)		
NAC	−0.22	0.5 mM (experimental 1)	Hydrophilic	[28,32]
		10 mM (experimental 2)		
GSH	−0.24	0.8 mM (serum)	Hydrophilic	[28,33,34]
		2–10 mM (intracellular)		

^1^ Oxidation reduction potential expressed in milli volts. The values provided are under standard conditions (25 °C, pH 7).

## Data Availability

The data presented in this study are available on request from the corresponding author.

## Supplementary Materials

The following supporting information can be downloaded at: https://www.mdpi.com/article/10.3390/ijms241713162/s1.
