# Supplementary material for: Effect of Antioxidant Supplementation on NET Formation Induced by LPS In Vitro; the Roles of Vitamins E and C, Glutathione, and N-acetyl Cysteine
Source: Int J Mol Sci. 2023 Aug 24;24(17):13162. doi: 10.3390/ijms241713162 (PMC10487622; doi:10.3390/ijms241713162)
Supplement: Supplementary file 1 [file ijms-24-13162-s001.zip › ijms-2574168-supplementary.pdf]

Supplementary Material

Flow cytometry apoptosis assay using Annexin V and PI staining to assess cellular apoptosis levels

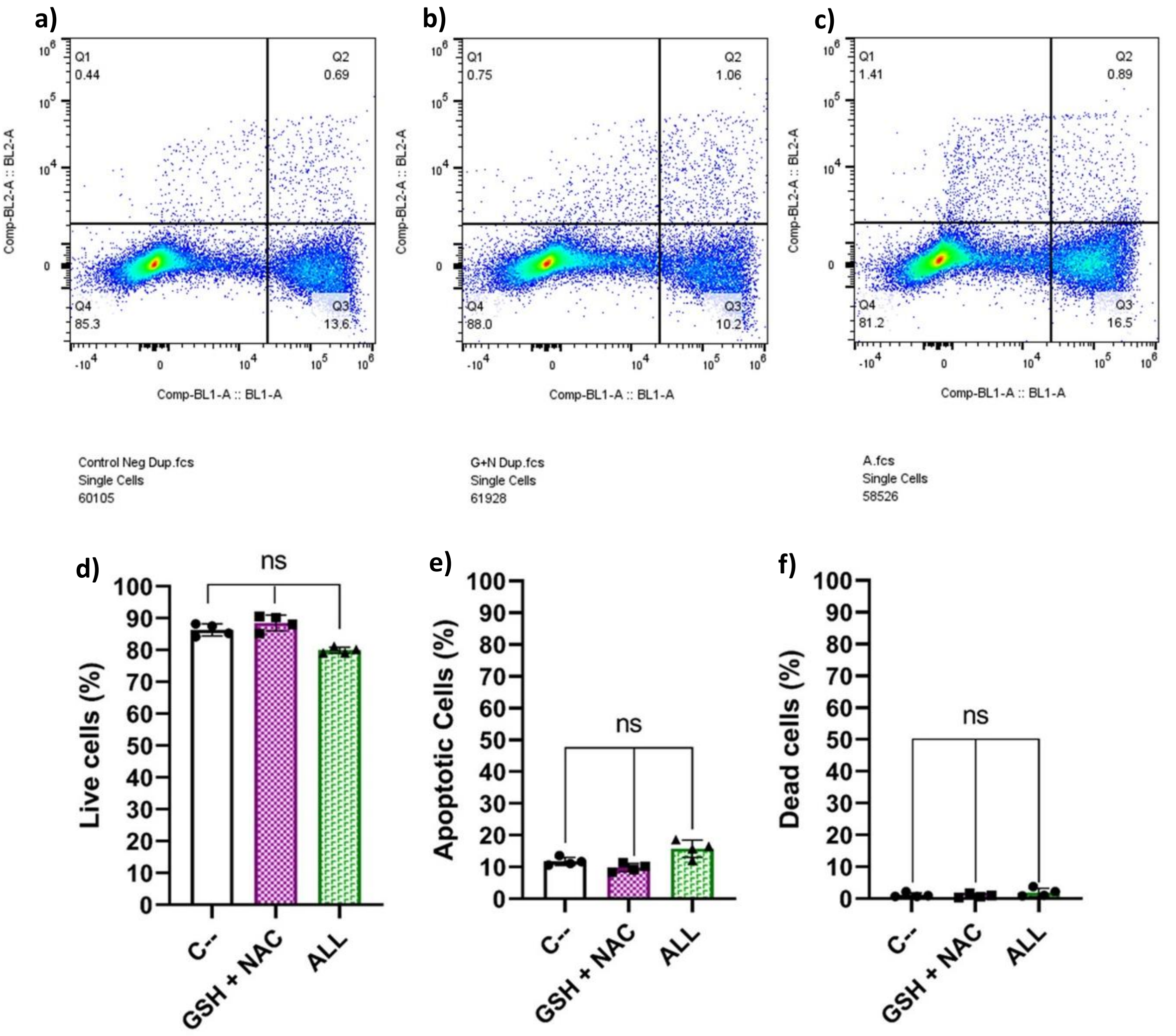

Figure S1. Antioxidant supplementation with GSH + NAC and ALL does not induce apoptosis in neutrophils. Freshly isolated neutrophils were supplemented with and without the antioxidant combinations GSH + NAC and ALL and incubated for 60 minutes. Apoptosis was assessed by flow cytometry using annexin V (BL1-A) and propidium iodide (BL2-A). a, b, and c) Representative dot-plot analysis for apoptosis assessment in neutrophils. The Q1 and Q2 represent the dead cells, the Q3 the apoptotic cells and the Q4 the live cells. d) Percentage of live neutrophils with and without antioxidant supplementation. e) Percentage of apoptotic neutrophils with and without antioxidant supplementation. f) Percentage of dead neutrophils. A total of 20,000 events were acquired, and the sample size consisted of 4 subjects analyzed in duplicates. Statistical analysis was performed using the Friedman test. Results are presented as mean ± standard deviation SD.

Cytotoxicity assay using the MTT Cell proliferation kit

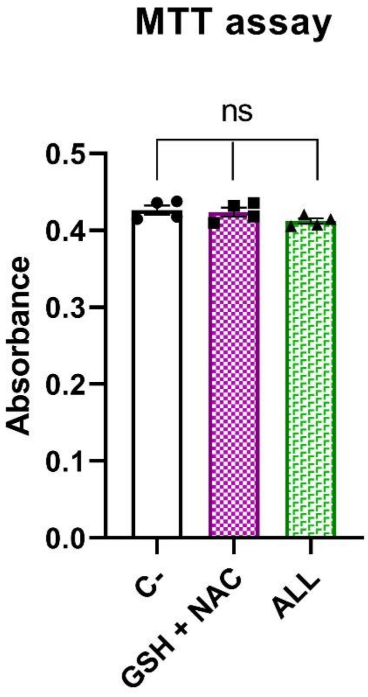

Figure S2. Antioxidant supplementation with GSH + NAC and ALL does not induce cytotoxicity in neutrophils. Freshly isolated neutrophils were supplemented with and without the antioxidant combinations GSH + NAC and ALL and incubated for 60 minutes. Cytotoxicity was assessed by measuring the conversion of water-soluble MTT into insoluble formazan by viable cells. The formazan crystals were subsequently solubilized with DMSO, generating a colored solution whose intensity is proportional to the concentration. The absorbance of the negative control and treated neutrophils showed no statistically significant difference. The sample size consisted of 4 subjects, and independent experiments were analyzed in triplicates. Statistical analysis was performed using one-way ANOVA followed by T3 Dunnett post hoc test. The results are presented as mean ± standard error (SE).

Total Antioxidant Capacity (TAC) assay of media used

a)

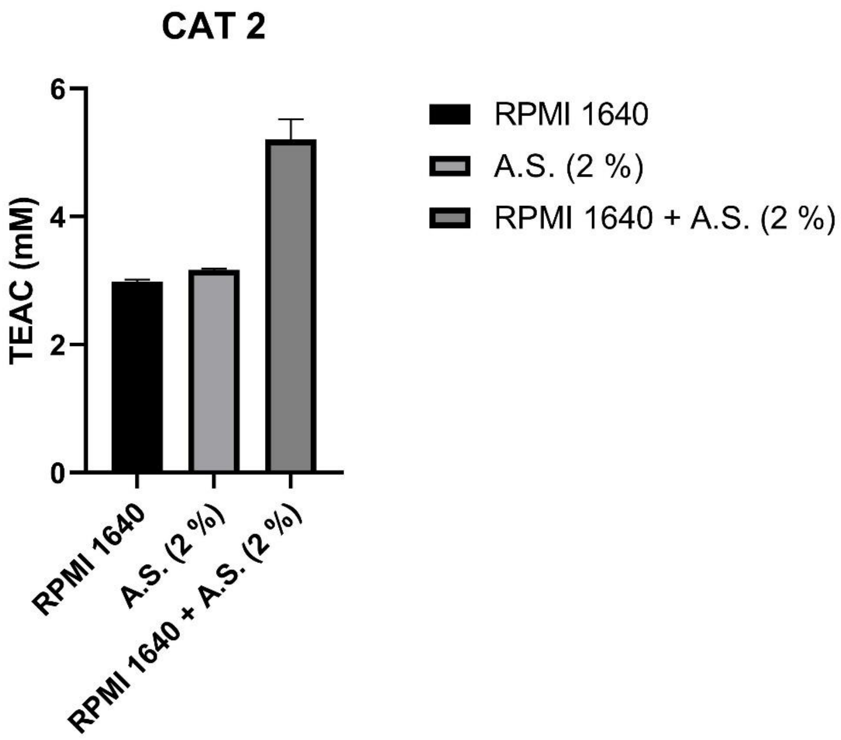

b)

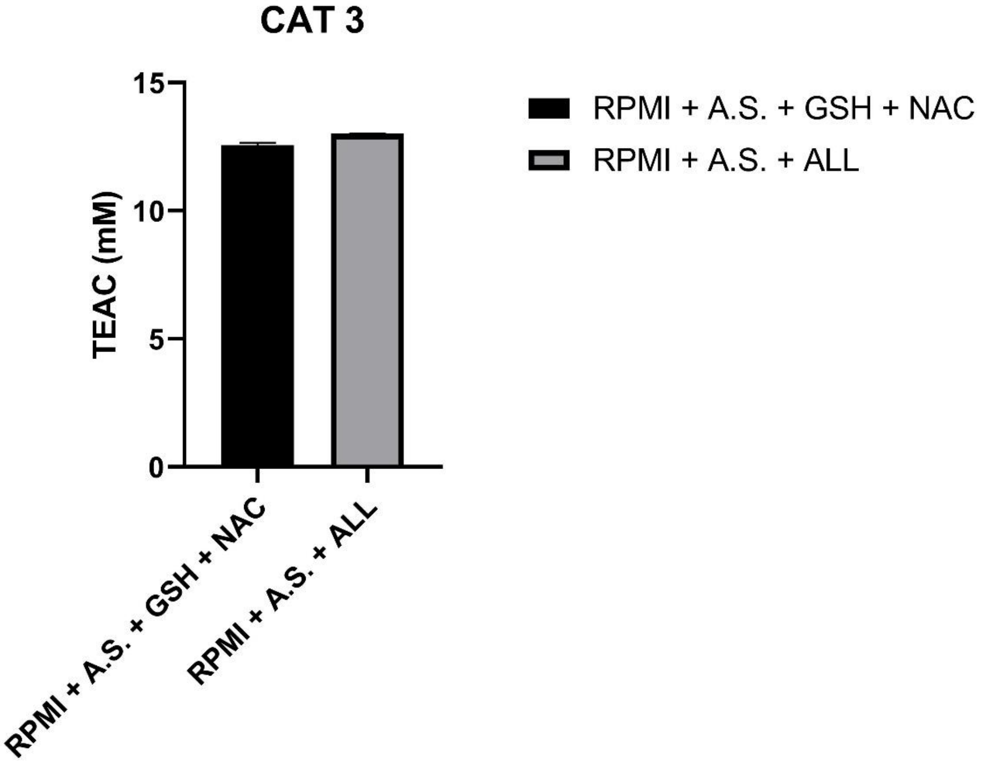

Figure S3. Total Antioxidant Capacity assay of supplemented and non-supplemented culture media. a) The TAC of the individual media components, namely RPMI 1640, AS 2%, and RPMI 1640 + AS 2%, was measured independently. b) The TAC of RPMI 1640 + AS 2% supplemented with two different antioxidant combinations, GSH + NAC and ALL, was determined. The experiment was performed with one triplicate of each condition. The antioxidants were added to the media according to the instructions provided in the corresponding section of the materials and methods. The results are reported as the mean ± standard error (SE) of millimoles (mM) of trolox equivalent antioxidant capacity (TEAC).

ROS Kinetics assessment during NET formation

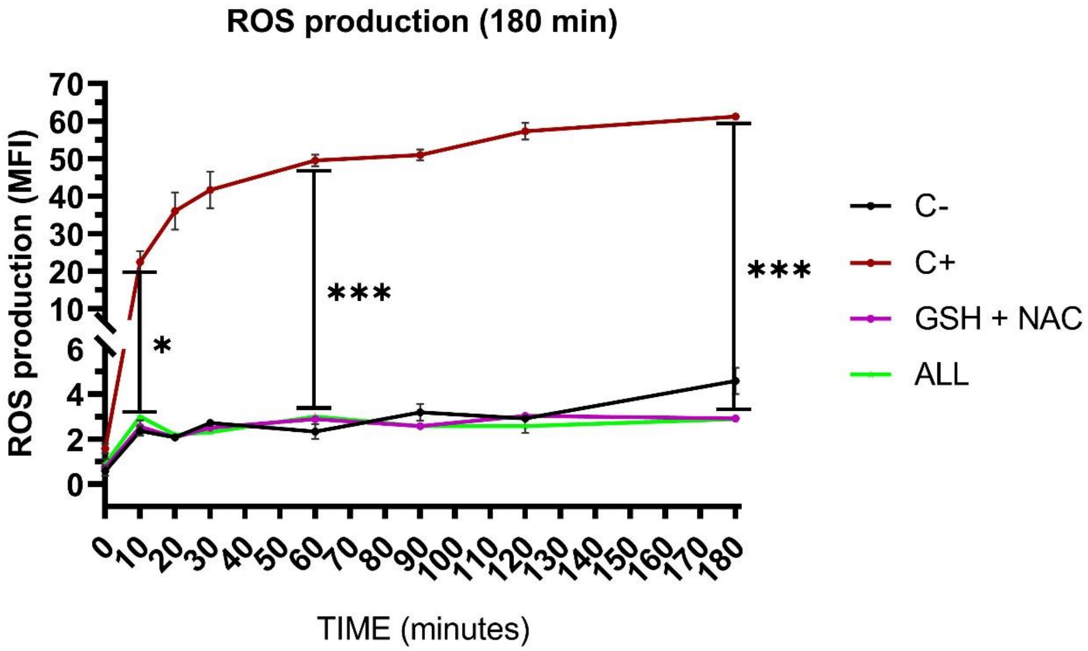

Figure S4. ROS production in stimulated neutrophils during NET formation. To assess ROS production, freshly isolated neutrophils incubated with the DCFDA probe and monitored for 180 minutes each 2 minutes. All samples contain 1.5 X 10^5 neutrophils per well in RPMI 1640 sodium bicarbonate buffered media (pH 7.3) + 2 % AS. Positive control and antioxidant groups (GSH + NAC and ALL) were stimulated with LPS. The time 0 corresponds to the initial measurement after neutrophil isolation and incubation with the DCFDA probe. The sample size was 4 subjects for each triplicate. Statistical analysis was performed analyzing the area under the curve (AUC) and performing one-way ANOVA and T3-Dunnet post hoc test. Results are presented as mean ± S.E. of median fluorescence intensity (MFI). Significant p values: < 0.03 (*), < 0.001 (***).
